# Draft Genome Sequence of *Clavibacter michiganensis* subsp. *nebraskensis* Strain DOAB 397, Isolated from an Infected Field Corn Plant in Manitoba, Canada

**DOI:** 10.1128/genomeA.00768-15

**Published:** 2015-07-09

**Authors:** James Tabi Tambong, Renlin Xu, Zaky Adam, Morgan Cott, Karin Rose, Lana M. Reid, Fouad Daayf, Stephan Brière, Guillaume J. Bilodeau

**Affiliations:** a Eastern Cereal and Oilseed Research Center (ECORC), Agriculture and Agri-Food Canada, Ottawa, Ontario, Canada; b Manitoba Corn Growers Association, Carman, Manitoba, Canada; c Department of Plant Science, University of Manitoba, Winnipeg, Manitoba, Canada; d Canadian Food Inspection Agency, Ottawa, Ontario, Canada

## Abstract

In 2014, the pathogen *Clavibacter michiganensis* subsp. *nebraskensis* was isolated from symptomatic corn leaves in Manitoba, Canada. We report the draft genome sequence of *C. michiganensis* subsp. *nebraskensis* DOAB 397, consisting of 3.059 Mb with 73.0% G+C content, 2,922 predicted protein-coding sequences, 45 tRNAs, 3 rRNAs, and 37 pseudogenes.

## GENOME ANNOUNCEMENT

*Clavibacter michiganensis* subsp. *nebraskensis* (CMN) is the causal agent of Goss’s wilt and blight disease of corn. The disease was first reported in 1969 in south-central Nebraska, USA, but became sporadic after partially resistant cultivars were identified in field corn (1). Goss’s wilt has reemerged and is spreading rapidly in major corn-growing regions of the United States and Canada. This pathogen is a concern to corn growers given the potential high yield losses (up to 50%) that could be incurred from systemic infections as well as its phytosanitary risk to nonendemic international trade partners such as Europe and China. In 2014, we isolated *Clavibacter michiganensis* subsp. *nebraskensis* DOAB 397 from symptomatic corn leaves in Manitoba, Canada, confirming the establishment of the disease in this Canadian province. We report the draft genome sequence of *Clavibacter michiganensis* subsp. *nebraskensis* DOAB 397. Genomic DNA shearing, library preparation, and the whole-genome shotgun sequence of *Clavibacter michiganensis* subsp. *nebraskensis* DOAB 397 were performed by the Génome-Québec Innovation Centre (Montreal, Canada). The draft genome was determined by paired-end sequencing using Illumina MiSeq technology (Génome-Québec, Montreal, Canada). A total of 1,078,908 paired-end reads, each 250 bp in length, totaling 269,727,000 bp, were obtained. The quality of the reads was checked by using FastQC version 0.11.3 (2). *De novo* assembly was performed using ABySS version 1.5.2 (3) at different *k*-mer values (75–113). A *k*-mer value of 97 gave the best assembly quality (no misassembled scaffolds) based on QUAST version 2.3 (4). Scaffolds with length <300 bp were discarded. The draft genome comprises 28 scaffolds (minimum, 3,207 bp; maximum, 428,173 bp; *N*_50_, 142,187 bp; total size, 3,059,794 bp). The G+C content of the draft genome is 73.0% with an overall estimated coverage of 89×.

The 28 scaffolds of *Clavibacter michiganensis* subsp. *nebraskensis* DOAB 397 were ordered and oriented based on the complete genome sequence of the type strain, *Clavibacter michiganensis* subsp. *nebraskensis* NCPPB 2581^T^ (NC_020891) using the CAR software (5). The NCBI Prokaryotic Genome Automatic Annotation Pipeline (PGAAP) revealed that the draft genome of *Clavibacter michiganensis* subsp. *nebraskensis* DOAB 397 contains 2,922 predicted protein-coding sequences, 45 tRNAs, 37 pseudogenes, 3 rRNAs, and 1 other RNA. Compared to PGAAP, CMG-Biotools (6) predicted 2,903 genes based on Prodigal (7), whereas RNAmmer version 1.2 (8) predicted 3 rRNAs containing one copy each of 16S rRNA, 5S rRNA, and 23S rRNA. The number of predicted tRNAs genes was confirmed by tRNAscan-SE version 1.3.1 software (9). Analysis of the draft genome of strain DOAB 397 revealed that it has 14 more genes than the complete genome sequence of *Clavibacter michiganensis* subsp. *nebraskensis* NCPPB 2581^T^, the type strain isolated in 1969. A more detailed analysis of strain DOAB 397 will be included in a future publication.

### Nucleotide sequence accession numbers. 

This whole-genome shotgun project has been deposited at DDBJ/EMBL/GenBank under the accession number LAKL00000000. The version described in this paper is the first version, LAKL01000000.

## References

[1] Agarkova IV, Lambrecht PA, Vidaver AK 2011 Genetic diversity and population structure of *Clavibacter michiganensis* subsp. *nebraskensis*. Can J Microbiol 57:366–374. doi:10.1139/w11-016. 21510777

[2] Andrews S 2015 FastQC: a quality-control tool for high-throughput sequence data. http://www.bioinformatics.babraham.ac.uk/projects/fastqc.

[3] Simpson JT, Wong K, Jackman SD, Schein JE, Jones SJ, Birol I 2009 ABySS: a parallel assembler for short read sequence data. Genome Res 19:1117–1123. doi:10.1101/gr.089532.108. 19251739 PMC2694472

[4] Gurevich A, Saveliev V, Vyahhi N, Tesler G 2013 QUAST: quality assessment tool for genome assemblies. Bioinformatics 29:1072–1075. doi:10.1093/bioinformatics/btt086. 23422339 PMC3624806

[5] Lu CL, Chen K-T, Huang S-Y, Chiu HT 2014 CAR: contig assembly of prokaryotic draft genomes using rearrangements. BMC Bioinformatics 15:381 doi:10.1186/s12859-014-0381-3. 25431302 PMC4253983

[6] Vesth T, Lagesen K, Acar Ö, Ussery D 2013 CMG-Biotools, a free workbench for basic comparative microbial genomics. PLoS One 8:e60120. doi:10.1371/journal.pone.0060120. 23577086 PMC3618517

[7] Hyatt D, Chen G-L, LoCascio PF, Land ML, Larimer FW, Hauser LJ 2010 Prodigal: prokaryotic gene recognition and translation initiation site identification. BMC Bioinformatics 11:119 doi:10.1186/1471-2105-11-119. 20211023 PMC2848648

[8] Lagesen K, Hallin P, Rødland EA, Staerfeldt HH, Rognes T, Ussery DW 2007 RNAmmer: consistent and rapid annotation of ribosomal RNA genes. Nucleic Acids Res 35:3100–3108. doi:10.1093/nar/gkm160. 17452365 PMC1888812

[9] Lowe TM, Eddy SR 1997 tRNAscan-SE: a program for improved detection of transfer RNA genes in genomic sequence. Nucleic Acids Res 25:955–964. doi:10.1093/nar/25.5.0955. 9023104 PMC146525

